# Human and feline adipose-derived mesenchymal stem cells have comparable phenotype, immunomodulatory functions, and transcriptome

**DOI:** 10.1186/s13287-017-0528-z

**Published:** 2017-03-20

**Authors:** Kaitlin C. Clark, Fernando A. Fierro, Emily Mills Ko, Naomi J. Walker, Boaz Arzi, Clifford G. Tepper, Heather Dahlenburg, Andrew Cicchetto, Amir Kol, Lyndsey Marsh, William J. Murphy, Nasim Fazel, Dori L. Borjesson

**Affiliations:** 1 Veterinary Institute for Regenerative Cures and Department of Pathology, Microbiology and Immunology, University of California, Davis, CA 95816 USA; 2 Institute for Regenerative Cures and Department of Cell Biology and Human Anatomy, University of California, Davis, CA 95816 USA; 3 Department of Surgical and Radiological Sciences, School of Veterinary Medicine, University of California, Davis, CA 95816 USA; 4 Department of Biochemistry and Molecular Medicine, University of California, Davis, CA 95816 USA; 5 Department of Dermatology, School of Medicine, University of California, Davis, CA 95816 USA

**Keywords:** Multipotent adult progenitor cell, Mesenchymal stem cell, Adipose tissue, Feline, Human, Animal model, Immunomodulation

## Abstract

**Background:**

Adipose-derived mesenchymal stem cells (ASCs) are a promising cell therapy to treat inflammatory and immune-mediated diseases. Development of appropriate pre-clinical animal models is critical to determine safety and attain early efficacy data for the most promising therapeutic candidates. Naturally occurring diseases in cats already serve as valuable models to inform human clinical trials in oncologic, cardiovascular, and genetic diseases. The objective of this study was to complete a comprehensive side-by-side comparison of human and feline ASCs, with an emphasis on their immunomodulatory capacity and transcriptome.

**Methods:**

Human and feline ASCs were evaluated for phenotype, immunomodulatory profile, and transcriptome. Additionally, transwells were used to determine the role of cell-cell contact in ASC-mediated inhibition of lymphocyte proliferation in both humans and cats.

**Results:**

Similar to human ASCs, feline ASCs were highly proliferative at low passages and fit the minimal criteria of multipotent stem cells including a compatible surface protein phenotype, osteogenic capacity, and normal karyotype. Like ASCs from all species, feline ASCs inhibited mitogen-activated lymphocyte proliferation in vitro, with or without direct ASC-lymphocyte contact. Feline ASCs mimic human ASCs in their mediator secretion pattern, including prostaglandin E2, indoleamine 2,3 dioxygenase, transforming growth factor beta, and interleukin-6, all augmented by interferon gamma secretion by lymphocytes. The transcriptome of three unactivated feline ASC lines were highly similar. Functional analysis of the most highly expressed genes highlighted processes including: 1) the regulation of apoptosis; 2) cell adhesion; 3) response to oxidative stress; and 4) regulation of cell differentiation. Finally, feline ASCs had a similar gene expression profile to noninduced human ASCs.

**Conclusions:**

Findings suggest that feline ASCs modulate lymphocyte proliferation using soluble mediators that mirror the human ASC secretion pattern. Uninduced feline ASCs have similar gene expression profiles to uninduced human ASCs, as revealed by transcriptome analysis. These data will help inform clinical trials using cats with naturally occurring diseases as surrogate models for human clinical trials in the regenerative medicine arena.

**Electronic supplementary material:**

The online version of this article (doi:10.1186/s13287-017-0528-z) contains supplementary material, which is available to authorized users.

## Background

Mesenchymal (multipotent) stem cells (MSCs) are proliferative, plastic adherent, fibroblast-like stromal cells that can be isolated from many tissue types [1]. Adipose tissue is an important tissue source for MSC expansion (ASCs) [2]. Fat collection is relatively noninvasive, the frequency of MSCs in fat is about 10-fold higher than in bone marrow, and fat is more readily available in small animals, older animals, and people. In comparative studies, ASCs appear to be highly enriched in immune-related genes and may have advantages in terms of proliferation, stability, and immunomodulatory ability, perhaps as a result of their retaining the metabolic and inflammatory modulating transcriptome native to its tissue [3–7].

Immunomodulation by MSCs represents a novel therapeutic option for various inflammatory and immune-mediated diseases [2, 8, 9]. MSCs modulate cell activation, differentiation, and secretion of almost all cells of the innate and humoral immune systems. MSCs inhibit T-cell proliferation with an associated decrease in tumor necrosis factor (TNF)α secretion in humans, rodents, and veterinary species including horses and dogs [8–10]. Human, equine, and canine ASCs secrete soluble mediators that are known to be immunomodulatory, including prostaglandin E2 (PGE_2_), indoleamine 2,3 dioxygenase (IDO), interleukin (IL)-6, IL-8, transforming growth factor (TGF)β, and vascular endothelial growth factor (VEGF) [6, 7, 11–14]. Human MSCs and ASCs also regulate T-cell and B-cell function via direct cell-cell interaction [15–17]. The increased recognition of the role of MSCs in immunomodulation, combined with successes in phase I–III clinical trials in humans using stem cell therapy for inflammatory and immune-mediated diseases, has resulted in recommendations that immune functional assays be developed as potency-release criteria for advanced-phase clinical trials [18].

Naturally occurring diseases in companion animals are increasingly recognized for the important role they play in discovery about human diseases and translational medicine [19]. These diseases in companion animals may better reflect the complex genetic, environmental, and physiological variations present in human diseases compared to induced diseases in laboratory animals [20]. Clinical trials in academic veterinary medicine mirror that of human healthcare systems, and animal owners are eager to enroll in these trials [20]. The selection of appropriate large animal models for preclinical testing in human stem cell trials should rely on solid comparative studies, as MSCs from rodents may or may not recapitulate the immunomodulatory profile of human MSCs. For example, there are important differences between human and murine MSC secretion profiles that may dictate how predictive mouse models are for studies that focus on MSCs and innate immune cells, CD8^+^ T cells, and mechanism-of-action studies [9, 21–23].

Companion animals can serve as relevant, preclinical, surrogate, translational models for MSC therapy and contribute directly to investigational new drug applications for human clinical trials. Cats with naturally occurring diseases analogous to human conditions can provide insight into feasibility, safety, and biologic activity of novel stem cell therapies. The development of appropriate and relevant large animal models will: 1) reduce the work needed when translating pre-clinical data to human trials; and 2) assist in choosing human trials to pursue. Naturally occurring diseases in cats can help determine the ideal route of cell administration, cell dose, the number of cell doses, and efficacy readouts to optimize the potential for successful human clinical trials. Cats develop a variety of spontaneous inflammatory and immune-mediated diseases, and stem cell therapy trials are already underway for chronic oral mucosa inflammation [24], asthma [25], and chronic enteropathy [26]. For example, feline chronic gingivostomatitis has a similar T-cell activation phenotype to the human oral inflammatory diseases oral lichen planus, apthous stomatitis, and pemphigus vulgaris [27, 28]. Cats also serve as valuable models for noninflammatory diseases, including chronic renal failure [29, 30].

The objectives of this work were to delineate a comprehensive immunomodulatory profile of feline ASCs and to perform a direct side-by-side comparison of feline and human ASCs as a first step in determining how ASCs from this relevant large animal species compare to human ASCs. We found that cat ASCs largely mimic human ASCs in basic phenotype, early proliferative ability, transcriptome, secretion profile (notably IDO and PGE_2_), and functional downregulation of activated T-cell proliferation.

## Methods

### Tissue samples

Human adipose tissue was obtained as discarded material from female patients undergoing a breast reduction procedure in compliance with an approved protocol (*n* = 5; University of California, Davis (UCD) Institutional Research Board, protocol number 258314-8). For all human tissues collected, informed patient consent forms were obtained. Subcutaneous feline adipose tissue was surgically obtained from specific pathogen-free (SPF) cats (*n* = 3) and from client-owned cats (*n* = 3) undergoing routine surgery. Fat collection was conducted according to a protocol approved by the Institutional Animal Care and Use Committee, and the Clinical Trials Review Board, UCD (protocol number 18422). All owners of client-owned cats signed an informed consent form. All cats were free of feline immune deficiency virus and feline leukemia virus infection.

### Human ASC isolation and expansion

Adipose tissue (~20 g) was mixed with 10 mL phosphate-buffered saline (PBS) and homogenized (gentleMACS dissociator; Miltenyi Biotec, San Diego, CA, USA). Samples were centrifuged at 1500 rpm for 5 min and the cell/tissue pellet was incubated with 10 mL animal origin-free collagenase (CLSAFA, 267 U/mL; Worthington Biochemical, Lakewood, NJ, USA) for 30 mins with gentle agitation at 37 °C. Samples were centrifuged at 2000 rpm for 7 min and cell pellets were resuspended and plated at 1000 cells/cm^2^ in plastic culture flasks using standard culture medium (minimum essential medium (MEM)α; GE Healthcare Life Sciences HyClone Laboratories, Logan, UT, USA) supplemented with 10% fetal bovine serum (FBS; Atlanta Biologicals, Flowery Branch, GA, USA), 1% l-glutamine, and 1% penicillin/streptomycin (ThermoFisher Scientific, Gibco, Pittsburgh, PA, USA). After 2 days, nonadherent cells were removed by washing twice with PBS. MSCs from passages 3–5 were used for experimentation.

### Feline ASC isolation and expansion

ASCs were isolated from fat and cultured as previously described [24]. ASCs from passages 3–5 were used for experimentation.

### ASC morphology and size

Adherent feline and human ASCs were imaged on a phase-contrast microscope using bright field. To quantify cell sizes, images were taken of five random areas of two human and two feline ASC lines grown in T225 flasks. Twenty cells from each image were measured using ImageJ software.

Comparison of cell size in suspension of human (*n* = 2) and feline (*n* = 2) ASCs was assessed by flow cytometry. Human ASCs were labeled using CD105 conjugated with APC (BD Biosciences, Pharmingen, San Jose, CA, USA) and feline ASCs were labeled using CD90 conjugated with PE (Leukocyte Antigen Biology Laboratory, UCD, clone CA1.4G8) as previously described [24, 31]. Labeled human and feline lines were mixed 1:1 and read on a flow cytometer (Cytomics FC500; Beckman Coulter, Brea, CA, USA). Cell size of fluorochrome-labeled human and feline cells was determined by forward and side scatter.

### Karyotype

Feline (*n* = 2, passage 5) and human (*n* = 2, passage 4) ASCs were karyotyped as previously described [32]. ASCs were plated in a six-well plate, treated with colcemid (ThermoFisher Scientific) to arrest the mitotic cells in metaphase, lifted and prepared with serial washes and fixations for staining and microscopic analysis. Analysis included scanning all slides, counting a minimum of 20 metaphases, analysis of a minimum of seven metaphases, and karyotyping a minimum of two metaphases.

### ASC surface phenotype

Human and feline ASCs (*n* = 5 each) were incubated for 45 min with fluorophore-conjugated antibodies including CD44, CD45 (human ASCs), CD18 (feline ASCs), CD90, CD105, and MHCII as previously described [24, 31]. All human antibodies were purchased from BD Biosciences and feline antibodies were purchased from the Leukocyte Antigen Biology Laboratory, UCD, unless otherwise indicated (MHC II (clone 42.3), CD18 (clone FE3.9 F2), CD90 (clone CA1.4G8), CD44 (clone IM7; BioLegend, San Diego, CA, USA), and CD105 (SN6; eBioscience, SanDiego, CA, USA). Mouse IgG-APC (MCA928; AbD Serotec, Kidlington, Oxford, UK) was used as an isotype control. All samples were run on a flow cytometer (Cytomics FC500). Flow cytometry data were analyzed using FlowJo flow cytometry software (Tree Star, Ashland, OR, USA).

### Osteogenesis

Osteogenic assays were performed as previously described [33, 34]. In brief, 10,000 ASCs/cm^2^ were cultured for 14 days with media changes every 3 days (*n* = 2 human ASCs and *n* = 2 feline ASCs). Osteogenic medium consisted of standard culture medium supplemented with 0.2 mM ascorbic acid (Sigma-Aldrich, St. Louis, MO, USA), 0.1 μM dexamethasone (Sigma-Aldrich), and 20 mM β-glycerolphosphate. Cells were fixed with 10% vol/vol formalin solution for 15 min, washed once with PBS, and stained for 20 min with 1% wt/vol Alizarin Red S (ARS, Ricca Chemical Company, Arlington, TX, USA) with gentle shaking to stain for precipitated calcium (Sigma-Aldrich). Samples were then photographed.

### ASC proliferation

ASCs (*n* = 4 human, *n* = 5 feline) were plated into two T25 flasks at 5000 cells/cm^2^. At each passage, ASC viability was determined (trypan blue exclusion dye; ThermoFisher Scientific) and ASCs were enumerated (by hemocytometer). ASCs were passaged at 70–80% confluence. In order to normalize differences in when cells were passaged, population doubling times were calculated as previously described [35]. Cultures were terminated at passage 6.

### Lymphocyte suppression assay (LSA)

LSAs were run, with and without a transwell, to compare the ability of feline and human ASCs to inhibit activated T-cell proliferation and to determine if T cell-ASC contact is necessary for inhibition of lymphocyte proliferation (*n* = 5 feline and *n* = 5 human lines). Our goal was to maximally stimulate human and feline T-cell proliferation using mitogens. Previous work in our laboratory in horses [14], dogs, and cats (unpublished data) demonstrated that allogenic peripheral blood mononuclear cells (PBMCs) only weakly and variably stimulate T-cell proliferation. Our pilot data demonstrated, in agreement with others, that concanavalin A (ConA) is the best and most potent mitogen for mature, circulating, post-thymic cells in cats (unpublished data and [36–38]) whereas leucoagglutinin (PHA) is the best T-cell mitogen for human T lymphocytes [39, 40].

Feline PBMC isolation and LSAs were carried out as previously described [14, 24]. In brief, PBMCs were isolated from whole blood using gradient centrifugation and were co-incubated with irradiated ASCs in culture wells at a 1:5 (PBMC:ASC) ratio and activated with 5 mg/mL ConA (Sigma-Aldrich). Cells were co-cultured for 4 days. Control wells included PBMCs alone, ASCs alone, ConA-stimulated PBMCs, and PBMCs mixed with ASCs without ConA stimulation. To determine the role of contact, cells were plated in transwell dishes (Corning 0.4 μM polycarbonate membrane 24-well plate; Corning, NY, USA) with PBMCs in the bottom and ASCs in the insert. To determine IDO activity, the experiment was run as described; however, media were supplemented with l-tryptophan (Sigma-Aldrich) to a final concentration of 600 μM.

For human LSAs, PBMCs were isolated using Ficoll-Paque (GE Healthcare) according to the manufacturer’s instructions and MSC-PBMC co-incubation, with and without transwells, was performed as previously described for feline LSAs except the medium used was αMEM (GE Healthcare Life Sciences) with 16% FBS (Atlanta Biologicals), 1% penicillin/streptomycin (ThermoFisher Scientific), and 1% Glutamax (ThermoFisher Scientific), and the PBMCs were stimulated with PHA (Sigma-Aldrich; 2.5 μg/ml) [39, 40].

Human and feline PBMC proliferation was measured via 5-bromo-29-deoxyuridine (BrdU) incorporation (BrdU Flow Kit; BD Biosciences) and analyzed on a flow cytometer (Cytomics FC500). Flow cytometry data were analyzed using FlowJo flow cytometry software (Tree Star).

### Mediator secretion (ELISA)

Feline and human PGE_2_, TGFβ1, VEGF, interferon (IFN)γ, TNFα, IL-6, IL-8, and IL-10 were measured in LSA supernatants in duplicate using enzyme-linked immunosorbent assay (ELISA) kits following the manufacturer’s instructions. Feline analytes were measured with feline-specific kits (IFNγ, TNFα, IL-6, IL-8, and IL-10: Duosets; R&D Systems, Minneapolis, MN, USA) or feline-validated kits (TGFβ1: multispecies TGF-β1 (ThermoFisher Scientific) [41]; VEGF: human QuantiKine Kit (R&D) [42, 43]; and PGE_2_: competitive ELISA (Enzo Life Sciences, Farmingdale, NY, USA) [44, 45]). Human TGFβ1, VEGF, IFNγ, TNFα (QuantiKine), PGE_2_, (competitive ELISA kit), IL-6, IL-8, and IL-10 (DuoSets) were all purchased from R&D. All ELISA samples were read on a Synergy HTMulti-Mode microplate reader with Gen5 software (Biotek, Winooski, VT, USA).

### IDO assay

IDO catalyzes the conversion of tryptophan to N-formyl kynurenine, which is then catabolized to kynurenine. Kynurenine levels are directly proportional to IDO activity. Two volumes of LSA media (that had been supplemented with tryptophan) were treated with 1 volume of 30% trichloroacetic acid (Sigma-Adrich) and centrifuged. Equal parts of trichloroacetic acid-treated supernatant and Ehrlich’s reagent (1% *p*-dimethylaminobenzaldehyde in glacial acetic acid; Sigma-Aldrich) were mixed and read at 490 nm on a microplate reader (Synergy HT Multi-Mode Gen5 software) [14].

### RNA isolation

Total RNA was isolated from three primary feline ASC cultures using the RNeasy Mini Kit (Qiagen, Inc., Valencia, CA, USA). RNA quantity and quality were assessed on a NanoDrop spectrophotometer (Thermo Scientific) and the Agilent 2100 Bioanalyzer (Agilent Technologies, Santa Clara, CA, USA), respectively.

### RNA-Seq library preparation and next-generation sequencing (NGS)

Whole transcriptome profiling was performed using a directional, strand-specific mRNA-Seq approach for three feline ASC lines. Briefly, total RNA samples were submitted to the UC Davis Comprehensive Cancer Center’s Genomics Shared Resource (GSR), and indexed RNA-Seq libraries were prepared from 200 ng total RNA using the KAPA Stranded mRNA-Seq Kit (Kapa Biosystems, Inc., Wilmington, MA, USA) according to the manufacturer’s standard protocol. Poly-adenylated mRNA was purified from total RNA and ribosomal RNA removed by binding to oligo (dT) beads, which was followed by RNA fragmentation by incubation at 94 °C in the presence of magnesium. Double-stranded cDNA was then generated by random-primed first-strand synthesis and subsequent second-strand synthesis in the presence of dUTP for strand marking. The double-stranded cDNA was then 3’-A tailed and indexed, Illumina-compatible adapters were ligated. The libraries were then enriched by high-fidelity PCR amplification (15 cycles) with KAPA HiFi HotStart DNA Polymerase and adapter-specific primers. Subsequently, libraries were combined for multiplex sequencing on an Illumina HiSeq 4000 System (100-bp, paired-end; ~30 million reads/sample) [46].

### Next generation sequencing data analysis

Image processing, base calling, quality scoring (Phred), and sample demultiplexing were executed by HiSeq Control Software with Real Time Analysis (HCS v3.3.41/RTA 2.5.2) and bcl2fastq Conversion Software (Illumina; San Diego, CA, USA). FASTQ-formatted sequence data were analyzed using a standard HISAT (hierarchical indexing for spliced alignment of transcripts)-Cufflinks workflow. RNA-Seq sequence reads (FASTQ format) were aligned to the reference cat genome assembly (Nov. 2014, ICGSC Felis Catus 8.0) using HISAT software [47]. Gene- and transcript-level expression were comprehensively quantified with Cufflinks software [48], which performed: 1) transcript assembly; 2) identification of splice variants; 3) quantification of expression as FPKM (fragments per kilobase of transcript per million mapped reads) values; and 4) normalization. Normalized FPKM values (Cuffnorm output) were utilized for downstream analysis steps. Statistical analyses and hierarchical clustering of the data were performed with GeneSpring GX software (Agilent Technologies, Inc.), and gene-annotation enrichment analysis performed with tools available from the Database for Annotation, Visualization and Integrated Discovery (DAVID v6.7) [49, 50].

Meta-analysis of the feline ASC expression data was performed with RNA-Seq data for human ASCs (NCBI GEO Accession GSE37521) [51]. For this, gene expression values from both datasets (feline ASCs: FPKM; human ASCs: RPKM (Reads Per Kilobase of transcript per Million mapped reads)) were separately normalized (quantile), joined on similar Gene IDs, and then baseline-transformed. Values for replicate samples were averaged and, subsequently, hierarchical clustering (similarity measure: Euclidean; linkage rule: centroid) was performed on the genes exhibiting variance across the human ASC cell types (≥2-fold change relative to undifferentiated ASCs).

### Statistical analyses

For all cell-based assays, normal distribution of the data was tested using the Kolmogorov and Smirnov test. For normally distributed data, a one sample Student’s *t* test (normalized data; lymphocyte proliferation) or paired *t* test (non-normalized data) or analysis of variance (ANOVA; >2 comparisons) was used. For feline non-normally distributed data, a Mann–Whitney-Wilcoxon test was used to determine differences in protein secretion data. Human data were analyzed using Wilcoxon matched pairs test. Human inflammatory mediators were normalized to paired lymphocyte donors before analysis was performed. Commercially available statistical software was used for all statistical analyses (GraphPad InStat version 3.06 for Windows; GraphPad, La Jolla, CA, USA). Results are presented as mean and standard error. A *P* value of <0.05 was considered statistically significant.

## Results

### Human and feline ASCs are morphologically and phenotypically similar

**Fig. 1:**
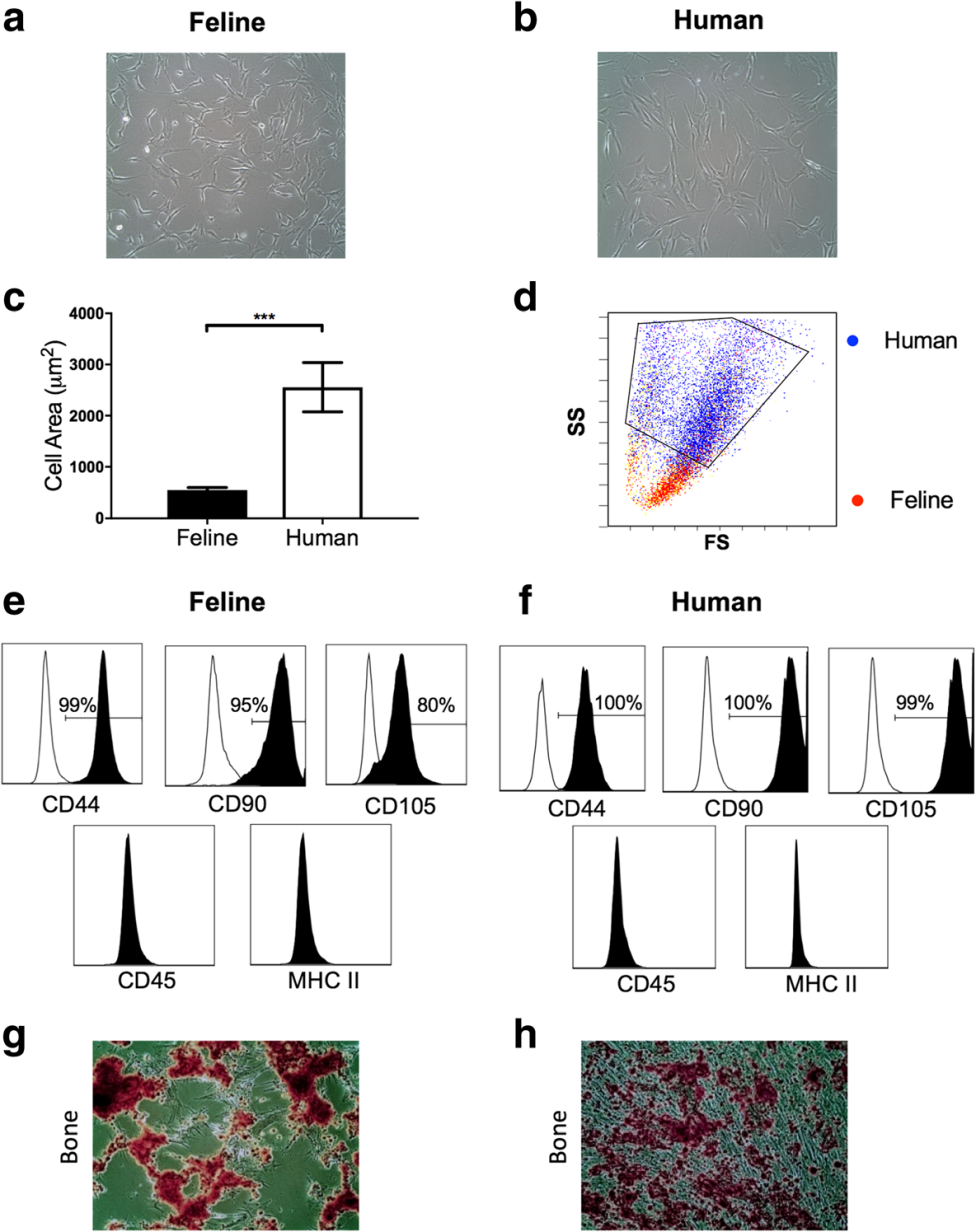
Human and feline ASCs possess typical MSC characteristics. Feline (**a**) and human (**b**) ASCs adhere to plastic and have a spindle, fibroblast morphology in culture. However, human ASCs are significantly larger than feline ASCs when adherent (**c**) and in suspension (**d**). Feline (**e**) and human (**f**) ASCs both have positive surface expression of CD44, CD90, and CD105, and are negative for surface expression of CD45 (pan leukocyte, human) or CD18 (pan leukocyte, feline) and MHC II. Both feline (**g**) and human (**h**) ASCs undergo osteogenic differentiation. Cell size presented as mean and standard error. *P < 0.05, **P < 0.01, ***P < 0.001

The ASCs derived from feline and human adipose tissue had typical spindle-shaped, adherent morphology (Fig. 1a and b). However, human ASCs were significantly larger, both when adhered to plastic and when in suspension, than feline ASCs (adherent cells, *p* < 0.001, Fig. 1c; suspended ASCs, Fig. 1d). Both feline and human ASCs had a normal metaphase spread and karyotype (Additional file 1: Figure S1). The surface protein expression on feline and human ASCs was compared using markers that define MSCs [1]. Both feline and human ASCs were strongly positive for CD44, CD90, and CD105, and were negative for leukocyte markers (CD18 (feline) and CD45 (human)), and MHCII (Fig. 1e and f) [31, 52, 53]. Both feline and human ASCs were capable of osteogenic differentiation (Fig. 1g and h). Others have established the full trilineage differentiation capacity of feline ASCs, including chondrogenic and adipogenic differentiation [52, 54–57]. Together, these data suggest that feline ASCs are karyotypically normal, meet the minimal criteria of multipotent stem cells and are smaller but otherwise morphologically and phenotypically identical to human ASCs.

### Human and feline ASCs are highly proliferative and inhibit allogeneic mitogen activated T-cell proliferation

**Fig. 2:**
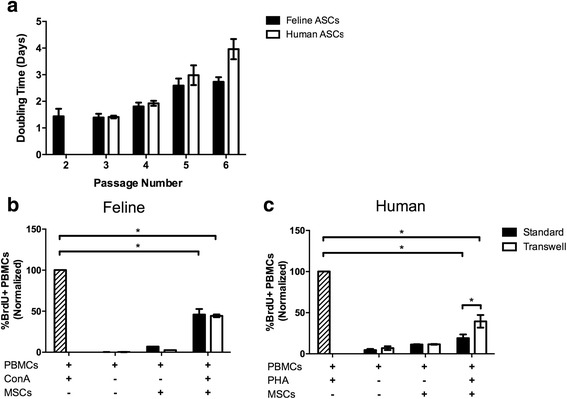
Human and feline adipose-derived mesenchymal stem cells (*ASCs*) display similar proliferative capacity and immune-suppressive functions. Doubling times were calculated for feline ASCs at passages 2–6 and for human ASCs at passages 3–6. The proliferation rate of human and feline ASCs is comparable throughout multiple passages (**a**). Feline ASCs co-incubated with stimulated peripheral blood mononuclear cells (*PBMCs*) suppress lymphocyte proliferation regardless of direct contact (transwells) (**b**). Human ASCs similarly inhibit leucoagglutinin (*PHA*)-induced lymphocyte proliferation; however, direct contact with stimulated PBMCs leads to stronger inhibition than the transwell condition (**c**). Data presented as mean and standard error. *P < 0.05, **P < 0.01, ***P < 0.001. *BrdU* 5-bromo-29-deoxyuridine, *ConA* concanavalin A, *MSC* mesenchymal stem cell

Human and feline ASCs readily proliferated in culture with comparable doubling times from passages 3–6 (clinically relevant passage numbers; Fig. 2a). Previous data from our laboratory and others has demonstrated a significant slowing of feline ASC proliferation after passage 5 [31, 52]. Human and feline ASCs significantly reduced activated T-cell proliferation (*P* < 0.004 all conditions compared to positive control; Fig. 2b and c). This inhibition occurred with or without ASC-T cell contact. However, human ASCs, when in direct contact with activated T cells, more potently reduced activated T-cell proliferation as compared to human ASCs plated in transwells (*P* = 0.03; Fig. 2c). These findings suggest that feline ASCs, like other species, are potent modulators of activated T-cell proliferation and that this modulation is largely mediated by secreted factors. Interestingly, human ASCs also markedly decrease activated T-cell proliferation, but this regulation is stronger when ASCs are in direct contact with T cells.

### Activated human and feline ASCS secrete high concentrations of immunomodulatory mediators

**Fig. 3:**
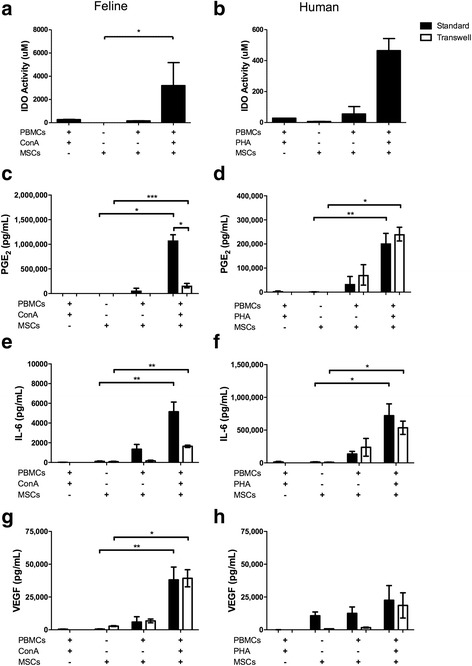
Human and feline ASCs produce immunomodulatory mediators. Feline ASCs in the presence of proliferating peripheral blood mononuclear cells (*PBMCs*) increase indoleamine 2,3 dioxygenase (*IDO*) activity (**a**). Increased IDO activity was observed by stimulated human ASCs, but was not statistically significant (**b**). Production of prostaglandin E_2_ (*PGE*_*2*_) occurred in both standard and transwell conditions in cats; however, greater production occurred when feline ASCs were in direct contact with stimulated PBMCs (**c**). Human ASCs produce PGE_2_ to the same magnitude with or without contact to stimulated PBMCs (**d**). Interleukin-6 (*IL-6*) was significantly upregulated regardless of contact for both feline (**e**) and human (**f**) ASC co-cultures. Feline ASCS produced vascular endothelial growth factor (*VEGF*) in standard and transwell conditions in cats (**g**). However, VEGF was not significantly increased above baseline by stimulated human ASCs (**h**). Data presented as mean and standard error. *P < 0.05, **P < 0.01, ***P < 0.001. *ConA* concanavalin A, *MSC* mesenchymal stem cell, *PHA* leucoagglutinin

ASCs function in large part via the secretion of mediators that regulate cells of the cellular and humoral immune system. We measured a number of mediators implicated in the immunomodulatory function of ASCs in parallel feline and human assays, with and without activation, and with or without cell-cell contact, to define feline ASCs and dissect out similarities and dissimilarities between cat and human ASCs. ASCs of both species variably secrete very low concentrations of IDO, PGE_2_, IL-6, and VEGF at baseline in culture, or in the context of allogeneic PBMCs; however, ASCs in the context of mitogen-activated T cells secrete significantly higher concentrations of immunomodulatory mediators. Activated feline ASCs secreted high concentrations of IDO (Fig. 3a), similar to canine and human MSCs (Fig. 3b) but unlike murine MSCs [21]. Activated feline ASCs also secrete high concentrations of PGE_2_ (Fig. 3c) that, unlike human ASCs (Fig. 3d), is significantly augmented by ASC-T cell contact. Activated feline and human ASCs secrete high concentrations of IL-6 (Fig. 3e and f) and VEGF (Fig. 3g and h), with or without MSC-T cell contact. Human ASC secretion of VEGF was not enhanced by T-cell activation (Fig. 3h).

**Fig. 4:**
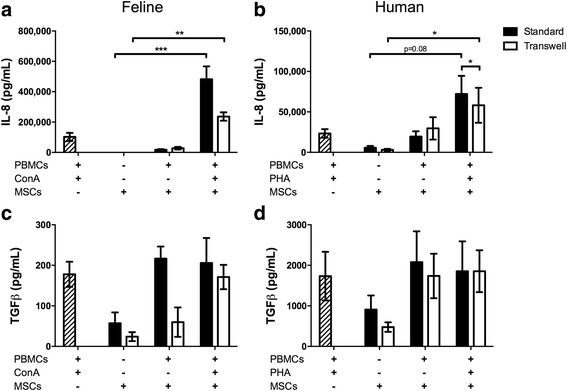
Human and feline ASCs increase interleukin-8 (*IL-8*) but not transforming growth factor beta (*TGFβ*) above baseline peripheral blood mononuclear cell (*PBMC*) production. Production of IL-8 by feline ASCs is significantly increased regardless of contact with stimulated PMBCs (**a**). IL-8 is also produced by stimulated human ASCs with (*P* = 0.08) and without contact (**b**). Production of TGFβ by activated mesenchymal stem cells (*MSCs*) is not altered from baseline expression regardless of contact in both cats (**c**) and humans (**d**). Data presented as mean and standard error. *P < 0.05, **P < 0.01, ***P < 0.001. *ConA* concanavalin A, *PHA* leucoagglutinin

We measured two mediators, IL-8 and TGFβ, that are potentially secreted by both activated PBMCs and ASCs (Fig. 4). For both feline and human cells, more IL-8 is present in activated ASC-PBMC co-cultures than is present in cultures with ASCs alone (Fig. 4a and b; *P* = 0.08) regardless of cell-cell contact. Cell-cell contact significantly increased IL-8 secretion for human cultures (*P* < 0.05) but not for feline cultures (*P* = 0.12). Feline TGFβ secretion mimicked our findings for human ASCs. Regardless of species, TGFβ was secreted by activated PBMCs, by ASCs at baseline, and in the context of ASC-PBMC co-incubation, with or without activation and with or without contact (Fig. 4c and d). The concentrations varied between conditions; however, there was no condition which significantly altered TGFβ secretion.

Findings agree with previous data from horses, humans, and dogs in which TGFβ is constitutively secreted and not, by definition, upregulated by activation [14, 58, 59].

**Fig. 5:**
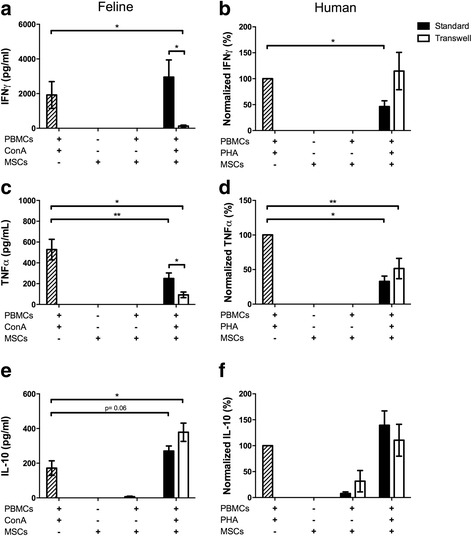
Regulation of inflammatory mediators by human and feline ASCs differs. Feline ASCs in the presence of proliferating lymphocytes do not inhibit production of interferon gamma (*IFNγ*). Removal of direct contact between ASCs and stimulated peripheral blood mononuclear cells (*PBMCs*) led to a significant reduction in IFNγ production (**a**). Human ASCs in contact with PBMCs stimulated with leucoagglutinin (*PHA*) inhibit production of IFNγ. However, when contact with activated PBMCs is removed, inhibition of IFNγ does not occur (**b**). Feline ASCs inhibit production of tumor necrosis factor alpha (*TNFα*) with or without contact, with the transwell condition inhibiting production of the mediator to a greater extent (**c**). Human ASCs inhibit production of TNFα regardless of contact with proliferating lymphocytes (**d**). Production of interleukin-10 (*IL-10*) in feline ASC lymphocyte co-cultures increased significantly without contact and was also increased with contact (*P* = 0.06) (**e**). No alterations in IL-10 production were observed by activated human ASCs (**f**). Data presented as mean and standard error. *P < 0.05, **P < 0.01, ***P < 0.001. *ConA* concanavalin A, *MSC* mesenchymal stem cell

Activation of PBMCs results in the secretion of IFNγ, TNFα, and IL-10; however, neither feline nor human ASCs, even in the context of allogenic PBMCs, secreted these mediators (Fig. 5). Typically, the inhibition of lymphocyte proliferation is associated with decreased IFNγ and TNFα production and variable IL-10 secretion. Unlike human ASCs, feline ASCs only inhibited IFNγ secretion in the absence of direct MSC-T cell contact whereas, when ASCs were in contact with T cells, IFNγ was not inhibited. Conversely, human ASCs were capable of inhibiting IFNγ secretion when ASCs and T cells were in direct contact; however, in the absence of contact, ASCs were not able to inhibit IFNγ (Fig. 5a and b). Both feline and human ASCs inhibited TNFα secretion; however, feline ASCs were more able to inhibit TNFα, like IFNγ, when ASCs were not in direct contact with T cells (Fig. 5c and d). Activated feline ASCs did increase PBMC secretion of IL-10; however, activated human ASCs did not alter IL-10 secretion regardless of cell-cell contact (Fig. 5e and f).

### Transcriptome analysis of feline ASCs demonstrates their relatedness and insight into their biological properties

**Fig. 6:**
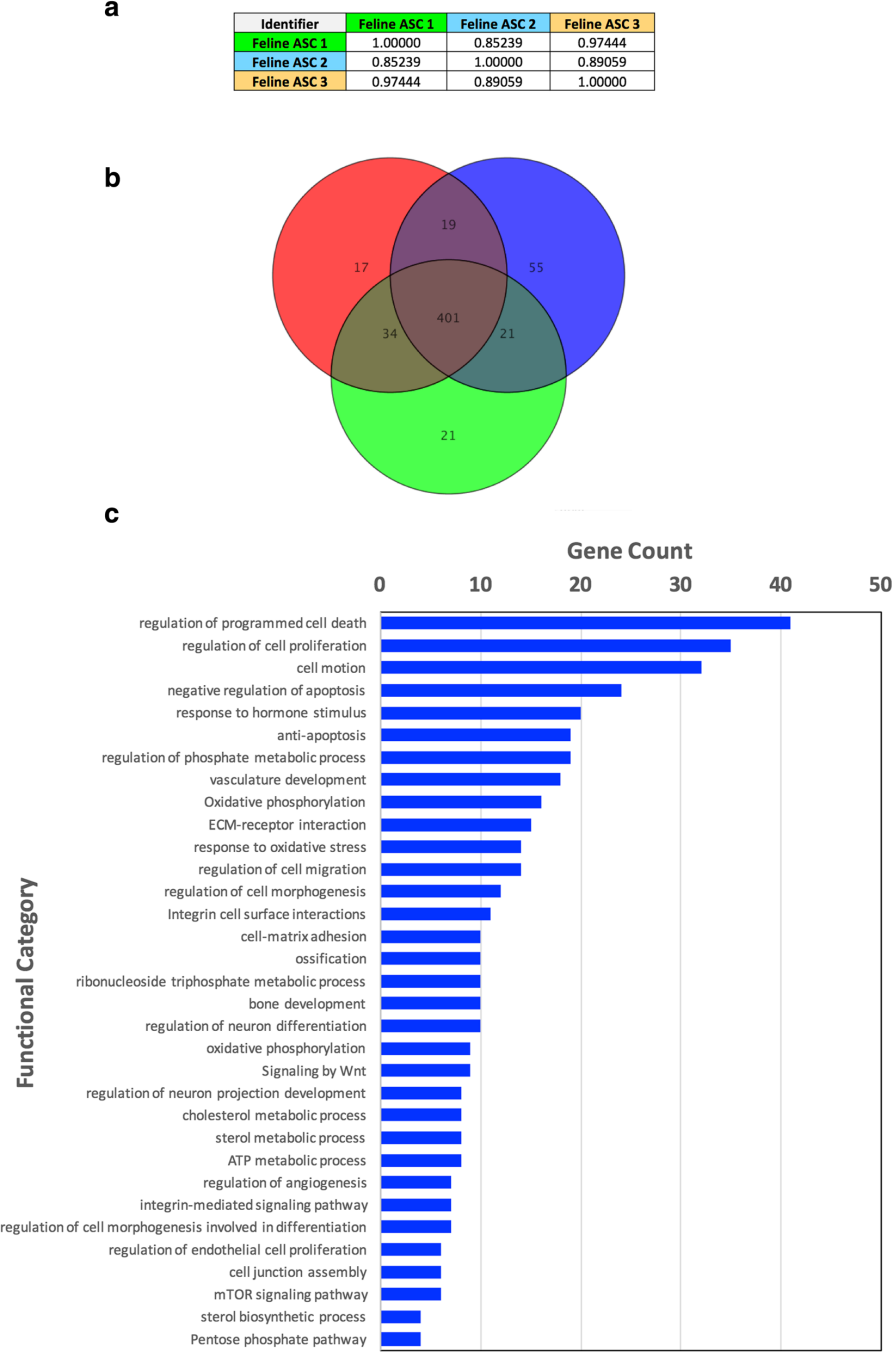
Transcriptome analysis reveals similarities between human and feline adipose-derived mesenchymal stem cells (*ASCs*) at the molecular level. Total RNA was isolated from undifferentiated feline ASC primary cultures established from three different cats. Correlation coefficients (Pearson *r*) show the relatedness of the different feline ASC cultures as determined by pair-wise correlation analysis of the gene-level FPKM data (**a**). Substantial similarity of the feline ASC lines is demonstrated by the Venn diagram, which depicts a greater than ≥87% overlap in gene expression profiles (401 genes) based on all genes exhibiting a high level of expression (≥100 FPKM) (**b**). Functional analysis reveals prominent biological processes in feline MSCs. Gene annotation-enrichment analysis of the most highly expressed genes (≥100 FPKM) in common to the three ASC preparations was performed. The gene ontology and pathway annotations (biological processes and pathways) specifically enriched within this gene list were filtered for statistical significance (Fisher Exact *P* value ≤0.1), and selected, representative categories presented (**c**)

Having defined and compared the biological features of feline ASCs, transcriptome analysis was next performed in order to evaluate the relatedness of the feline ASCs to each other, as well as to human ASCs, and to gain insight into the functional implications of the ASC gene expression profile (full dataset in Additional file 2: Figure S2; available at NCBI GEO Datasets Accession number: GSE94773. Pair-wise correlation analyses of the gene-level expression data (i.e., FPKM) for each ASC culture demonstrated that there was a high level of similarity between the ASC preparations isolated from the three cats (Pearson *r* = 0.853, 0.891, and 0.974) (Fig. 6a). There was a significant overlap of genes (2743 genes, 89.00–90.47%) that exhibited at least a moderate level of expression (i.e., ≥15 FPKM) in each ASC preparation. Significant overlap of gene expression profiles from the three feline ASC lines was highly expressed in 401 genes (≥100 FPKM; Fig. 6b). In order to gain insight into the functional significance of the ASC expression repertoire, functional annotation analysis was performed on the most highly expressed genes (i.e., ≥100 FPKM; 401 genes) that were common to all ASCs, with the notion that this would highlight the most prominent processes. For this, the gene set was analyzed with tools available from DAVID Bioinformatics Resources 6.7 that evaluate gene-gene functional relationships and enrichment for biological “modules” [49, 50]. There was over-representation of gene ontology categories involved in a number of processes, including: 1) the regulation of apoptosis; 2) cell adhesion; 3) response to oxidative stress; 4) regulation of cell differentiation; and 5) various metabolic processes, including phosphate metabolism, sterol metabolism, and oxidative phosphorylation (Fig. 6c).

**Fig. 7:**
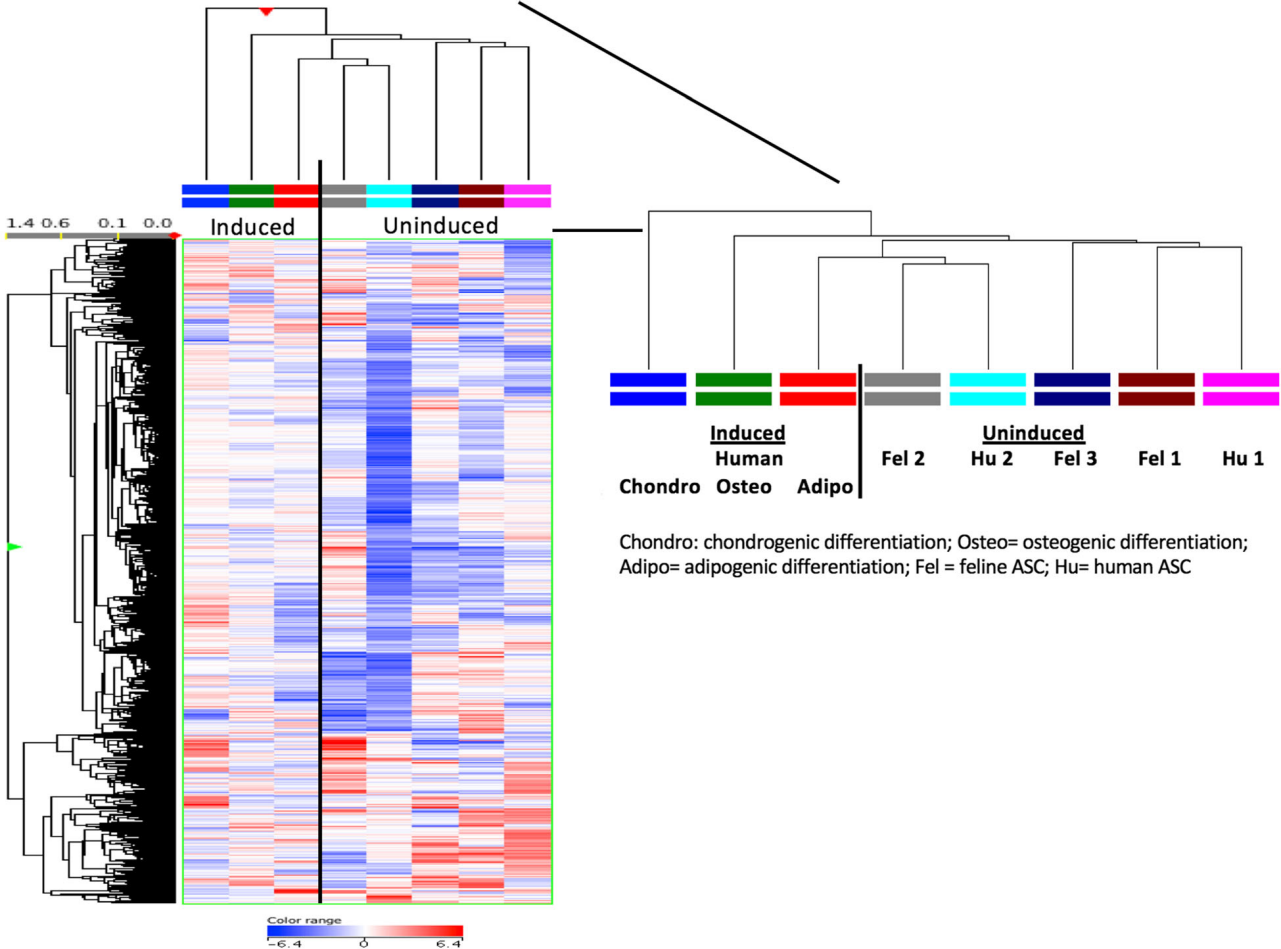
Noninduced feline and human adipose-derived mesenchymal stem cells (*ASCs*) have similar gene expression profiles. Meta-analysis of feline ASC RNA-Seq data was performed with a dataset for human ASCs (NCBI GEO Accession GSE37521). Comparison analyses were performed to identify expression profiles distinctive for undifferentiated (noninduced) and differentiated ASCs. Induced and uninduced cell lines are separated as indicated by a vertical line. Clustering of the differentially expressed genes was performed and the results depicted in the heatmap for the three feline ASC and human ASC cultures. Each sample column represents averaged values of replicates

Finally, the potential similarity of feline ASCs to their human counterparts was investigated by performing meta-analysis with an RNA-Seq dataset from human ASCs that were either undifferentiated (noninduced) or induced to differentiate along the adipogenic, osteogenic, and chondrogenic lineages (NCBI GEO Accession GSE37521; Fig. 7) [51]. Genes exhibiting variance across the human ASC cell types (≥2-fold change relative to undifferentiated ASCs) were determined and then used as the basis for hierarchical clustering with the feline ASCs. The feline ASCs clustered closely and were intermingled with the undifferentiated human ASCs, demonstrating their similar patterns of gene expression to the human ASCs, and providing additional evidence that the feline ASCs are representative of undifferentiated phenotype (Fig. 7). The variable gene expression patterns for the human and feline ASCs was highlighted by the fact that some human and feline ASC were clustered closer together than human to human or feline to feline ASCs. In addition, one undifferentiated human ASC line (hu-2) and one feline uninduced ASC line (fel-2) were more closely related to adipo-induced hASCs than to noninduced human and feline ASCs (Fig. 7).

## Discussion

Clinical trials with cell therapies are increasingly taking advantage of companion animals with naturally occurring diseases as relevant, surrogate models to support novel human investigational drug applications (IND) and veterinary investigational new animal drug applications (INAD) [19, 20, 30, 60]. Cats are a particularly important translational model for a number of diseases that are similar in cats and humans, including FIV (HIV) [61], T cell-mediated oral mucosa inflammation [24], chronic renal failure [29], asthma [25], chronic enteropathy [26], and osteoarthritis. The goal of this work was to profile feline ASCs and directly compare their phenotypic, cellular, and immunomodulatory profiles with human ASCs to deepen our understanding of MSC-based therapies as we move towards mechanism-of-action studies in animals and people. We found that feline ASCs, like human ASCs, can be defined as MSCs based on defined criteria (surface phenotype, differentiation potential, morphology, doubling time, normal karyotype) and that their transcriptome clusters closely with uninduced (undifferentiated) human ASCs. Feline ASCs inhibit mitogen-activated T-cell proliferation and their profile of secreted immunomodulatory mediators closely mimics that of human ASCs, dominated by IDO, PGE_2_, TGFβ, IL-6, and IL-8. Feline ASCs are highly activated and functional in the presence of the proinflammatory cytokines, IFNγ and TNFα, and, like human [6] and canine ASCs [12], their regulation of IFNγ is complex and likely dictated by at least two mechanisms, depending on whether or not the ASCs are in direct contact with activated PBMCs.

The data we generated from human ASCs largely mimicked data from studies that either compared human ASCs with MSCs from other sources, especially bone marrow, or studies that determined the immunomodulatory phenotype of human ASCs. Similar to others, we found that ASCs were able to inhibit activated T-cell proliferation with or without direct ASC-T cell contact [62, 63]; however, we also found that this inhibition was more marked when ASCs were in contact with activated cells. MSC-immune cell contact, especially in the context of both IFNγ and TNFα, has been shown to upregulate PD-1, VCAM-1, and ICAM-1 and this contact can then augment the production of soluble mediators, notably IDO [7, 21, 64]. Our data are also aligned with data from fetal membrane, or decidual, MSCs that also inhibit lymphocyte proliferation more profoundly in the presence of MSC-immune cell contact [64, 65]. Contact-dependent inhibition of immune cell function is thought to be more important for local immunosuppression [65]. In the absence of cell-cell contact, human ASCs were unable to inhibit IFNγ secretion and IL-8 production was decreased.

Unlike human and other veterinary species studied to date [11, 35], feline ASCs expanded in standard culture conditions do not proliferate robustly in vitro after passage 5 or 6 [31, 54]. This is in spite of the fact that, similar to horses [35] and humans [66], adipose tissue is still the tissue of choice for feline MSC therapy due to the ease of collection, tissue abundance, and faster growth kinetics [53]. The most likely consideration for this poor, late-passage proliferation is inadequate cell culture condition optimization. This phenomenon is independent of feline foamy virus (FFV) infection as it occurs in ASC lines from SPF cats as well as client-owned cats free of FFV infection [31]. Our group has not investigated cell culture optimization with media supplementation of growth factors, increased FBS concentration, or cytokine supplementation; however, optimization would likely improve growth kinetics. Creating working cell banks of autologous or allogeneic ASCs at passage 1 and expanding to doses of 5 × 10^6^ cells/kg (passages 2 or 3) for clinical use is readily achieved given the high number of ASCs obtained from small feline fat samples even without culture optimization.

MSCs derived from different tissues can vary substantially in size; for example, equine cord blood-derived MSCs are much larger than equine ASCs [67]. In this study, we found that ASCs in humans and cats differ in size, with feline ASCs being much smaller than human ASCs. Cell size differences are likely to be irrelevant for basic biological functions; however, recognizing cell size differences may inform species-specific tissue engineering protocols and scaffold features [68]. MSC size has also been implicated in determining pulmonary entrapment after intravascular cell administration [69].

The key mediators secreted by human and feline ASCs were similar. We did not attempt to compare absolute concentrations of secreted mediators as the mitogens selected (ConA and PHA) were different, the ELISA kits were different, and the degree of stimulation varied in the assays. Both PHA and ConA are nonspecific, potent T-cell mitogens. Mitogen selection and concentration was based on our early data examining proliferation kinetics and degree of activation as well as other published data in cats and humans [36–40]. Regardless, a few patterns are of interest. TGFβ1, VEGF and, to a lesser extent, PGE_2_ were the only mediators secreted to a measurable level by feline and human ASCs at baseline with no activation. The constitutive secretion of TGFβ1 concurs with previous findings in human [6, 7], equine [14], and canine [12] ASCs. TGFβ1 is involved in immune regulation by and differentiation of Foxp3^+^ regulatory T cells and Th17 cells, and may play an important part in MSC immunomodulation although it is not involved in the inhibition of lymphocyte proliferation [7, 12, 70]. Interestingly, VEGF secretion was not increased after mitogen stimulation in human ASCs; however, VEGF secretion did increase after feline ASC activation. It may be that VEGF secretion in human ASCs is more sensitive to hypoxic signals rather than T-cell mitogens [71].

There were a number of differences in how feline and human ASCs inhibited lymphocyte proliferation and in their secretion profiles, with and without PBMC contact. These data suggest that there are multiple different mechanisms by which human and feline ASCs regulate inflammatory (most notably IFNγ) and regulatory (IL-10) mediator secretion by activated PBMCs, one dependent on cell contact and one primarily mediated by soluble factors. Also, PGE_2_ secretion was markedly diminished in the absence of direct cell-cell contact for cats. Data from our in vivo studies suggest that induction of T regulatory cells may be important as a mechanism by which MSCs cure oral inflammatory disease in cats [24]. T regulatory cells classically require cell-cell contact to function; however, many soluble mediators, including PGE_2_, TGFβ1, and IL-10, can also play a role in the induction of regulatory T cells [72, 73]. Deciphering these mechanisms is critical as, although both cell-cell contact and soluble mediator production are likely important in vivo, there is at least one condition under which IFNγ secretion is not decreased. Sustained secretion of IFNγ may have important implications for particular clinical applications as IFNγ stimulates MSCs, derived from a number of tissues, to produce IDO, TGFβ, and HGF, to increase cell-cell signaling via the upregulation of PD-L1 and ICAM-1, and to generate regulatory CD8^+^ T cells [21, 62, 64, 65, 74–76]. These data are in agreement with data from our in vivo study where two cats with severe, chronic, oral inflammation that fully responded to ASC therapy (complete, sustained disease resolution) did so in the context of high serum concentrations of IFNγ [24].

Our findings suggest that uninduced feline ASCs are highly similar to human ASCs in their basic morphology, phenotype, and transcriptome. The goal of the transcriptome analysis was to determine the similarity of feline ASCs to human ASCs at the molecular level. As such, we focused the analysis on the expression of those genes that are highly relevant to MSC biology and did not compare the relatedness of culture-expanded feline ASCs to somatic feline tissues. It is likely that a number of feline ASC-expressed genes are shared across different feline tissues. Inclusion of biological controls (feline somatic tissues) would be needed to fully determine the stem-cell specificity of these data. Interestingly, the RNA-Seq data from one human ASC line, induced towards adipogenesis, was more closely aligned to one human and one feline ASC line that were uninduced. The origin of our MSCs from adipose tissue may recapitulate ASCs induced to differentiate along the adipose lineage more closely than those induced to differentiate towards bone or cartilage.

In summary, the activation of feline ASCs potentiates the secretion of a panel of immunomodulatory mediators that is comparable to human ASCs and human fetal decidual stem cells with one end result of decreasing lymphocyte proliferation. These data provide additional rationale for the use of naturally occurring diseases in cats as appropriate surrogate models for human regenerative medicine trials with MSCs when the diseases are also shown to be comparable. These data also provide the first published immunomodulatory (secretion) and transcriptome profile of feline ASCs from which further mechanism-of-action studies can result.

## Conclusion

These data provide the first comprehensive side-by-side evaluation of human and feline ASCs including phenotype, immunomodulatory profile, and transcriptome. Findings suggest that feline ASCs are multipotent mesenchymal stem cells that, when activated by IFNγ, modulate lymphocyte proliferation using soluble mediators that mirror the human ASC secretion pattern. Transcriptome analysis revealed similar gene expression profiles to uninduced human ASCs and set the stage for deeper investigations using cats as surrogate models for human cell therapy-based clinical trials.

## Additional files

Additional file 1: Figure S1. Representative karyotypes of feline and human ASCs. Both feline (A, B) and human (C, D) ASCs have a normal karyotype. (TIFF 19104 kb)
Additional file 2: Figure S2. Transcriptome analysis of ASCs. Full dataset of ASC gene expression profile. RNA-Seq analysis was performed on total mRNA prepared from three separate feline noninduced ASC culture preparations (passage 3), followed by data analysis with a standard HISAT-Cufflinks pipeline to yield gene expression values expressed as FPKM. (XLSX 1420 kb)

## Abbreviations

ASC: Adipose-derived mesenchymal stem cell
BrdU: 5-Bromo-29-deoxyuridine
ConA: Concanavalin A
ELISA: Enzyme-linked immunosorbent assay
FBS: Fetal bovine serum
FFV: Feline foamy virus
GSR: Genomics Shared Resource
IDO: Indoleamine 2,3 dioxygenase
IFN: interferon
IL: Interleukin
IND: Investigational drug applications
LSA: Lymphocyte suppression assay
MEM: Minimum essential medium
MSC: Mesenchymal stem cell
PBMC: peripheral blood monocular cell
PBS: Phosphate-buffered saline
PGE_2_: Prostaglandin E2
PHA: Leucoagglutinin
SPF: Specific pathogen-free
TGF: Transforming growth factor
TNF: Tumor necrosis factor
UCD: University of California, Davis
VEGF: Vascular endothelial growth factor
