# Supplementary figures and images for: Human and feline adipose-derived mesenchymal stem cells have comparable phenotype, immunomodulatory functions, and transcriptome
Source: Stem Cell Res Ther. 2017 Mar 20;8:69. doi: 10.1186/s13287-017-0528-z (PMC5360077; doi:10.1186/s13287-017-0528-z)

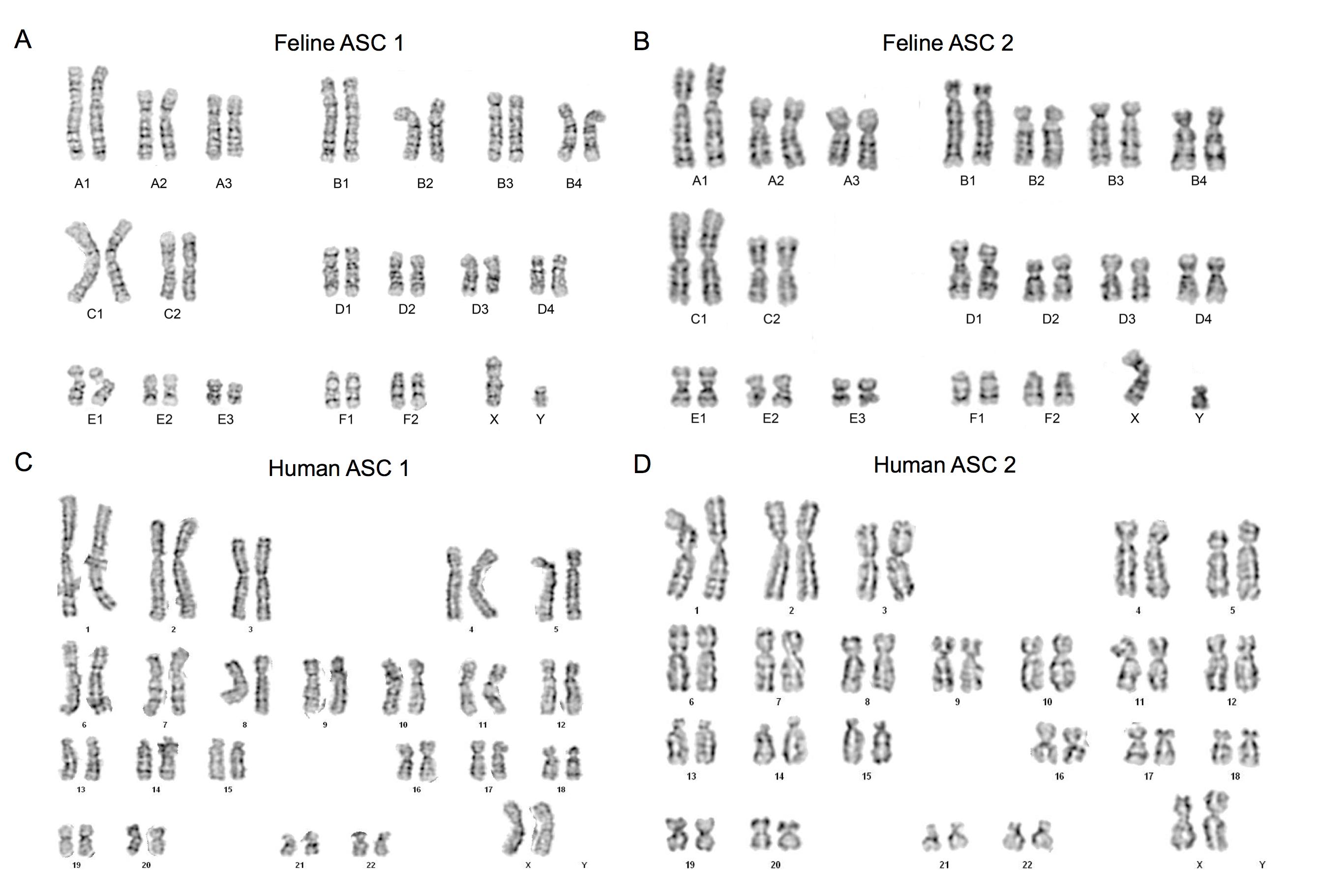

Supplement: Additional file 1: Figure S1. — Representative karyotypes of feline and human ASCs. Both feline (A, B) and human (C, D) ASCs have a normal karyotype. (TIFF 19104 kb) [file 13287_2017_528_MOESM1_ESM.tiff]
